# COVID-19 and vascular disease

**DOI:** 10.1016/j.ebiom.2020.102966

**Published:** 2020-08-20

Severe acute respiratory syndrome coronavirus 2 (SARS-CoV-2) was originally characterised as a novel respiratory coronavirus and was thought to primarily target pulmonary tissues in infected patients, similar to its close relative SARS-CoV, which was responsible for the epidemic of SARS in 2003. This preconception turned out to be an underestimation. Although SARS-CoV-2 does indeed infect pulmonary epithelial cells, it might also infect many other cell types, causing systematic inflammation with cytokine release and affecting multiple critical organs besides the lungs in severe cases.

In some patients, SARS-CoV-2 appears to attack the cardiovascular system, causing numerous cardiovascular complications. Back in January 2020, clinicians from Wuhan (Hubei, China) reported myocardial injury in patients with COVID-19 in a study published by *The Lancet*. In another study, published in *The Lancet Respiratory Medicine* on February 17, researchers observed interstitial mononuclear inflammatory infiltrates in the heart tissue of a deceased patient with COVID-19. Furthermore, myocardial damage and heart failure have been reported to contribute to causes of death that were linked to COVID-19 complications. In addition to inducing an overreactive inflammatory response, recent studies have shown that SARS-CoV-2 might also directly attack vascular endothelial cells and disrupt vascular barrier, leading to disseminated intravascular coagulation and inflammatory cell infiltration. As our understanding of the disease pathology improves, evidence is emerging that vascular pathology could have a substantial role in COVID-19 disease outcome.

In a *Lancet* paper published on April 20, 2020, Frank Ruschitzka and colleagues from University Hospital Zürich (Zürich, Switzerland) observed direct SARS-CoV-2 infection of endothelial cells and diffuse endothelial inflammation in vascular beds of different organs in patients with COVID-19. Indeed, the angiotensin converting enzyme 2 (ACE2) receptor required for SARS-CoV-2 infection is expressed on the surface of endothelial cells. Shortly after their study was published, several other post-mortem studies showed similar patterns of vascular damage in deceased patients who had COVID-19. For example, two studies published in *The New England Journal of Medicine* on May 21 and *The Lancet Respiratory Medicine* on May 27 showed distinctive vascular features of severe endothelial injury, widespread thrombosis with microangiopathy, and increased vascular angiogenesis in the lungs of patients with COVID-19. Thrombosis not only occurs in the infected lung, but also in other organs including the heart and kidneys, as Amy Rapkiewicz and colleagues reported in *EClinicalMedicine* on June 25. All these data indicate that vasculopathy is likely to be important in COVID-19 pathogenesis and endothelial cells could themselves have a role in orchestrating the destructive intravascular coagulopathy associated with SARS-CoV-2 infection.

Immune mechanisms have been proposed to explain COVID-19-associated intravascular coagulopathy. Injured endothelial cells cause vascular leakage, trigger uncontrolled blood clotting, and recruit different types of immune cells and immunological factors that result in widespread inflammation and further vascular damage, forming a vicious cycle. In a single-centre, cross-sectional study published in *The Lancet Haematology* on June 30, George Goshua and colleagues from Yale University (New Haven, CT, USA) determined the role of endotheliopathy in COVID-19-associated coagulopathy pathogenesis and provided novel mechanistic insights into COVID-19-associated endotheliopathy. The authors discovered increased concentrations of plasma von Willebrand factor (VWF) in patients with COVID-19, which increased with disease severity. Plasma concentrations of soluble thrombomodulin correlated with clinical outcomes, with in-hospital mortality significantly lower in patients with low soluble thrombomodulin than in patients with high soluble thrombomodulin. Only endothelial cells and megakaryocytes can produce VWF, which has a major role in blood coagulation. As commented by O'Sullivan and colleagues in the same issue, these data support a mechanistic model in which alterations of plasma VWF and thrombomodulin concentrations following endothelial cell injury caused by SARS-CoV-2 infection lead to clinical prothrombotic manifestations of coagulopathy in patients with COVID-19. In other words, released VWF binds to platelets, neutrophils, and monocytes to initiate microvascular thrombosis; meanwhile, thrombomodulin further promotes a procoagulant and proinflammatory local milieu within the injured vasculature.

An increasing appreciation of the role of endothelial cells in COVID-19 pathogenesis has prompted research into vascular normalisation and anticoagulation strategies. For instance, bevacizumab, a monoclonal antibody targeting VEGF, can inhibit its vessel-permeabilizing activity and could help to maintain vasculature integrity in patients with COVID-19. Clinical trials (NCT04344782, NCT04275414, and NCT04305106) are exploring the effect of targeting this vascular factor on COVID-19 disease. One Phase 3 trial from Canada (NCT04362085) is recruiting patients to test the effect of therapeutic anticoagulant heparin in patients who are hospitalised with COVID-19. We await results of these trials with hope.

Our understanding of COVID-19 has evolved rapidly over the past few months and new therapies have been proposed and tested. Nevertheless, many mysteries remain. Future scientific discoveries will shed new light on our understanding of COVID-19. In particular, we at *EBioMedicine* look forward to seeing translational and clinical studies that could accelerate the diagnosis, management and treatment of this devastating disease.

*EBioMedicine*

